# Evaluating metastatic risk in breast cancer through CTCs and L1CAM expression

**DOI:** 10.3389/fonc.2025.1686166

**Published:** 2025-11-11

**Authors:** Ni Liao, Qiong Guo, Jingjing Chen, Fulan Tan, Yan Huang, Jiansheng Yi, Yi Hu, Chen Zeng, Qianhui Ouyang, Zhouxi Chen, Wei Zhou

**Affiliations:** Department of Breast Surgery, Zhuzhou Hospital Affiliated to Xiangya School of Medicine, Central South University, Zhuzhou, China

**Keywords:** circulating tumor cells, epithelial-mesenchymal transition, L1 cell adhesion molecule, breast cancer, lymph node metastasis, prognostic biomarker

## Abstract

**Introduction:**

Circulating tumor cells (CTCs) and L1 cell adhesion molecule (L1CAM) are associated with breast cancer (BC) metastasis. This study investigated their potential as predictive biomarkers for lymph node metastasis in early-stage invasive breast cancer (ESIBC).

**Methods:**

Ninety-three ESIBC patients were enrolled. CTC phenotypes and L1CAM expression were detected in preoperative blood samples using the CanPatrol® CTC system and RNA-ISH. Associations with clinicopathological variables were analyzed.

**Results:**

CTCs were detected in 79.6% of patients. Hybrid CTCs (H-CTCs) and L1CAM-positive CTCs were significantly correlated with lymph node metastasis and Ki-67 expression. A nomogram integrating H-CTCs, L1CAM, and Ki-67 predicted metastatic risk with excellent accuracy (AUC = 0.98).

**Discussion:**

H-CTCs and L1CAM-positive CTCs serve as potential blood-based biomarkers for evaluating metastatic risk in BC.

**Conclusion:**

The combined detection of H-CTCs and L1CAM enhances preoperative prediction of lymph node metastasis and provides new insights into BC metastasis mechanisms.

## Graphical Abstract

**Figure:**
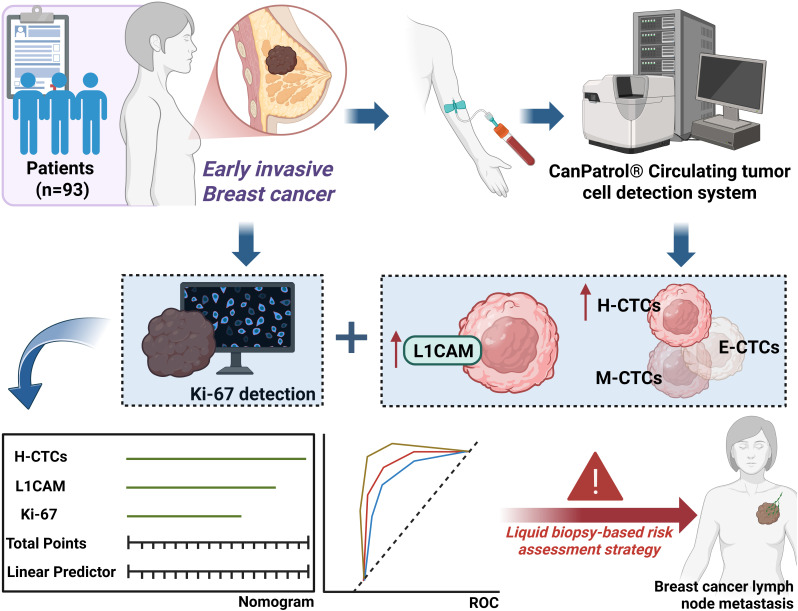
Schematic representation of a liquid biopsy strategy for evaluating metastatic risk in BC based on CTC subtypes and L1CAM expression.

## Highlights

- This study finds that the positivity rate of CTCs is significantly associated with tumor size and lymph node metastasis.
- The number of H-CTCs is significantly correlated with lymph node metastasis in BC, suggesting their potential predictive value.
- L1CAM shows a high positivity rate in CTCs, particularly in H-CTCs, and is strongly associated with tumor aggressiveness.
- A nomogram model combining H-CTCs, L1CAM, and Ki-67 is constructed to predict metastatic risk with high accuracy.
- This study provides novel molecular targets and predictive tools for assessing metastatic risk in BC.

## Introduction

Breast cancer (BC) is one of the most common malignancies among women worldwide, with an increasing incidence that poses a serious threat to women’s health and survival (1). Due to improvements in screening and treatment technologies, the overall survival rate of BC has improved; however, tumor heterogeneity and metastatic potential still place a subset of patients at risk for recurrence and poor prognosis (2). In particular, lymph node metastasis is a critical factor influencing staging, treatment strategies, and prognostic evaluation in BC. Accurate determination of lymph node involvement is therefore essential for clinical decision-making (3, 4). At present, lymph node metastasis is primarily assessed through imaging and intraoperative pathological biopsy, which are limited in sensitivity and fail to provide reliable preoperative individualized predictions (5–7). Consequently, the identification of more sensitive, non-invasive biomarkers capable of dynamic monitoring has become a key focus for improving early assessment of metastatic risk in BC.

Circulating tumor cells (CTCs), defined as tumor cells shed from primary or metastatic sites into the peripheral bloodstream, act as “seeds” in the process of distant metastasis (8). In recent years, CTCs have garnered increasing attention as a central component of liquid biopsy techniques, due to their potential for non-invasive and real-time monitoring through blood sampling (9, 10). Studies have shown that the clinical significance of CTCs lies not only in their quantity but also in their phenotypic heterogeneity, which is closely associated with tumor aggressiveness and metastatic capacity (11, 12). Based on their epithelial-mesenchymal transition (EMT) status, CTCs can be classified into three subtypes: epithelial (E-CTCs), mesenchymal (M-CTCs), and hybrid (H-CTCs) (13). H-CTCs, which exhibit both adhesion and migratory capabilities and represent an intermediate stage of dynamic EMT transformation, are considered the most metastasis-prone CTC subpopulation. Their presence has been strongly associated with increased metastatic risk across various solid tumors (14). In patients with primary BC, the presence of circulating tumor cells is also considered an independent adverse prognostic factor for disease-free survival, overall survival, breast cancer-specific survival, and distant disease-free survival (15). Therefore, recognizing the clinical significance of H-CTCs may enhance the precision of metastatic risk assessment in cancer patients.

L1 cell adhesion molecule (L1CAM) is a transmembrane glycoprotein belonging to the immunoglobulin superfamily and plays a crucial role in normal neural development (16, 17). In recent years, studies have shown that L1CAM is aberrantly overexpressed in various malignancies, where it regulates critical processes such as cell adhesion, migration, EMT, and chemoresistance (18, 19). In BC, elevated L1CAM expression has been strongly associated with high tumor grade, lymphovascular invasion, increased metastatic risk, and poor prognosis (20). Although its biological function has been investigated at the tissue level, the expression profile of L1CAM in peripheral blood CTCs and its relationship with EMT phenotypes remain unclear (21). Given the potential role of L1CAM in maintaining the EMT state of CTCs and promoting their invasive behavior, studies integrating CTC phenotyping and L1CAM expression may further elucidate the underlying mechanisms of metastasis in BC and enhance clinical predictive accuracy (22).

This study aimed to clarify the clinical and biological significance of CTCs in early-stage invasive breast cancer (ESIBC) by investigating the association between CTC phenotypic heterogeneity, L1CAM expression, and lymph node metastasis. We focused on the highly metastatic H-CTC subtype and its L1CAM expression pattern to explore its link with tumor aggressiveness. By identifying metastasis-relevant CTC subpopulations and molecular markers, our findings support the utility of liquid biopsy for early metastatic risk assessment and offer potential targets for anti-metastatic strategies in personalized breast cancer management.

## Materials and methods

### Study design

**Figure 1:**
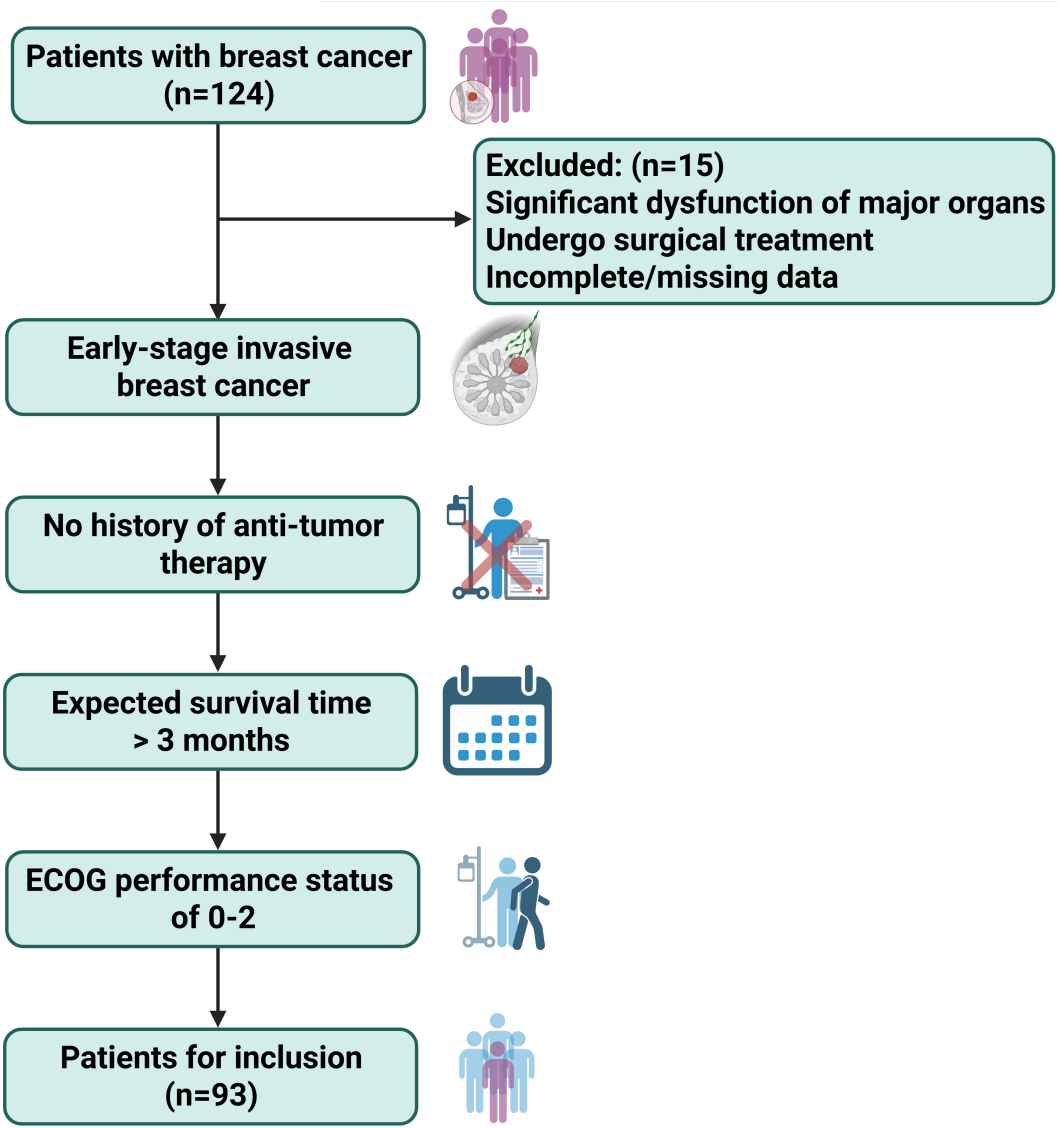
Flowchart of inclusion and exclusion criteria for patients with ESIBC to ISIBC.

This study was designed as a retrospective cohort study. The sample size estimation was based on previous literature and preliminary clinical pilot data, with the estimated positivity rate of CTCs set at approximately 30%. With a two-sided α of 0.05, an effect size of 0.3, and a statistical power (1-β) of 0.80, the minimum required sample size was calculated using PASS software (Version 15.0), resulting in 84 cases. Considering an estimated 10% risk of dropout or incomplete data, a total of 93 patients diagnosed with ESIBC at Zhuzhou Central Hospital between March 2019 and December 2021 were ultimately enrolled (**Figure 1**, **Supplementary Figure S1**). The inclusion criteria were as follows: (1) histologically confirmed ESIBC; (2) no prior history of cancer treatment; (3) an expected survival time of more than 3 months; and (4) an Eastern Cooperative Oncology Group (ECOG) performance status score of 0-2. The exclusion criteria included: (1) significant impairment of major organ function; (2) receipt of surgical treatment before enrollment; and (3) incomplete or missing clinical data. All patients provided written informed consent, and the study protocol was approved by the Ethics Committee of The Affiliated Zhuzhou Hospital of Xiangya Medical College Central South University.

### Observation indicators

The primary observation indicators of this study were the preoperative positivity rate of CTCs and the association between their EMT phenotypes—particularly H-CTCs—and lymph node metastasis in BC. Secondary indicators included estrogen receptor (ER), progesterone receptor (PR), and human epidermal growth factor receptor 2 (HER2) status; Ki-67 expression level; L1CAM expression in CTCs; and its correlation with molecular subtypes.

### Data collection

In this study, all clinical data were systematically collected by trained medical personnel before surgery. The information included patient age, ER status, PR status, HER2 status, molecular subtype, tumor size, TNM stage, lymph node metastasis status, and Ki-67 expression level.

The determination of ER and PR status was based on immunohistochemistry (IHC) results, with ≥1% of tumor cell nuclei exhibiting positive staining considered ER- or PR-positive. HER2 status was also assessed using IHC, with a score of 3+ interpreted as positive. Tumor molecular subtypes were classified according to the 2013 St. Gallen International Expert Consensus and relevant clinical guidelines, and were divided into five categories: (1) Luminal A: ER-positive, PR-positive (≥20%), HER2-negative, and low Ki-67 expression (<20%); (2) Luminal B (HER2-negative): ER-positive, PR-negative or low, HER2-negative, and high Ki-67 expression (≥30%); (3) Luminal B (HER2-positive): ER-positive, HER2-positive, regardless of PR or Ki-67 status; (4) HER2-overexpression: ER-negative, PR-negative, and HER2-positive; (5) Triple-negative: ER-negative, PR-negative, and HER2-negative. Lymph node metastasis was determined based on the 8th edition of the American Joint Committee on Cancer (AJCC) TNM staging criteria for BC, combined with intraoperative axillary lymph node dissection or sentinel lymph node biopsy and confirmed by postoperative pathological findings. The presence of cancer cell infiltration in regional lymph nodes was classified as lymph node metastasis, including macrometastasis (>2.0 mm), micrometastasis (0.2-2.0 mm), and isolated tumor cells (ITCs, <0.2 mm). For consistency in grouping, both micrometastases and ITCs were categorized as lymph node metastasis-positive in this study. Ki-67 expression was evaluated by IHC and expressed as the percentage of positively stained nuclei. A Ki-67 level of ≥30% was defined as high expression.

To ensure the objectivity and consistency of the data, all IHC-stained slides were independently evaluated in a blinded manner by two senior pathologists, without knowledge of the patients’ CTC test results. In cases of disagreement, a third experienced pathologist reviewed the slides and made the final judgment. Clinical data were independently extracted by dedicated data collectors based on pathology reports, examination results, and the hospital information system. The data collectors, laboratory personnel, and statistical analysts remained blinded to each other’s work, thereby maintaining the independence and scientific rigor of data collection and analysis.

### IHC

IHC was used to assess the expression of ER, PR, HER2, and Ki-67 antigen. BC tissue specimens were fixed in 10% neutral buffered formalin for 24 hours, routinely paraffin-embedded, and sectioned at a thickness of 4 μm. Staining was performed using the Ventana Benchmark XT automated IHC system (Roche). The primary antibodies used were as follows: ER (Roche, 790-4324), PR (Roche, 790-4296), HER2 (Roche, 790-2991), and Ki-67 (Roche, 790-4286). The staining procedure included deparaffinization, hydration, antigen retrieval, peroxidase blocking, incubation with primary antibody (37 °C for 30 minutes), DAB visualization, and hematoxylin counterstaining. Positive and negative controls were included in each batch to ensure the specificity and reliability of the staining results.

### Isolation, classification, and L1CAM detection of CTCs

CanPatrol^®^ CTC Analysis System (Model: CanPatrol CTC Analysis System, Manufacturer: SurExam Biotech, Suzhou, China) was used to isolate, enrich, classify, and detect the expression of L1CAM in CTCs from preoperative peripheral blood samples. A 5 mL peripheral blood sample was collected from each patient 1–3 days before surgery and stored in CTC-preservation tubes. Samples were processed within 4 hours of collection. Red blood cell lysis buffer was added to the samples and incubated at room temperature for 5–10 minutes to remove erythrocytes. The remaining cells were then enriched using an 8 μm pore-size nanomembrane filter, which retained the CTCs on its surface.

Following enrichment, the retained cells were fixed and pre-hybridized directly on the membrane. CTC phenotyping was subsequently performed using RNA *in situ* hybridization (RNA-ISH). SurExam Biotech provided probes targeting the following genes: epithelial markers (EpCAM, CK8, CK18, CK19), mesenchymal markers (Vimentin, Twist), a leukocyte exclusion marker (CD45), and L1CAM. All probes were used at a concentration of 250 nM. Hybridization was performed at 40°C for 2 hours using a matched hybridization buffer and a signal amplification system. Target signals were visualized using multicolor fluorescent labeling.

Under fluorescence microscopy, different types of CTCs exhibited distinct fluorescence characteristics: E-CTCs were CD45-negative and EpCAM/CK-positive, displaying red fluorescence; M-CTCs were CD45-negative and Vimentin/Twist-positive, showing green fluorescence; H-CTCs expressed both epithelial and mesenchymal markers and exhibited dual red and green fluorescence. L1CAM expression was detected using a custom RNA probe provided by the manufacturer, under hybridization conditions identical to those used for other probes. A positive L1CAM signal appeared as purple fluorescence (**Supplementary Figure S2**). Due to intellectual property protection, the L1CAM probe sequence was not disclosed; access could be requested from the manufacturer via a material transfer agreement (MTA). CTCs were classified as L1CAM-positive if at least two visible purple fluorescent signal dots were observed within the DAPI-stained nucleus, and the cell was CD45-negative with morphological features consistent with CTCs. L1CAM signals could be detected in E-CTCs, M-CTCs, or H-CTCs, and only signals with precise localization and minimal background interference were considered valid.

To ensure the accuracy and consistency of detection results, multiple quality control measures were implemented: (1) each assay batch included a positive control (BC cell line MCF-7) and a negative control (peripheral blood leukocytes from healthy donors); (2) all samples were tested in duplicate, with a concordance rate of ≥95% required between replicates; (3) two qualified technicians independently performed all experimental procedures; and (4) CTC enumeration and phenotypic classification were independently interpreted in a blinded manner by two investigators with intermediate or senior professional titles. In the event of a discrepancy, a third investigator reviewed the data, and the consensus result was included in the final statistical analysis.

### Statistical analysis

All statistical analyses were performed using SPSS version 26.0 (IBM Corp., USA). The normality of continuous variables was assessed using the Shapiro-Wilk test. Normally distributed data were expressed as mean ± standard deviation, while categorical variables were presented as frequencies (percentages). Between-group comparisons were conducted using the independent samples t-test. Multivariate logistic regression was used for regression analysis. A *p*-value < 0.05 was considered statistically significant.

## Results

### Demographic and clinical characteristics of patients

A total of 93 patients with ESIBC to intermediate-stage invasive breast cancer (ISIBC) who had not received any treatment before surgery were included in this study. Relevant demographic and clinical characteristics are summarized in **Supplementary Table S1**. The median age of the patients was 51 years, and over 85% were classified as TNM stage I-II. Hormone receptor positivity was relatively high, with Luminal A/B representing the predominant molecular subtypes, accounting for more than 60% of cases. Approximately one-third of the patients had lymph node metastasis, and overall Ki-67 expression levels were elevated. In general, the study population consisted primarily of middle-aged women with typical features of ESIBC.

### Distribution of CTC counts across clinical subgroups of BC

**Figure 2:**
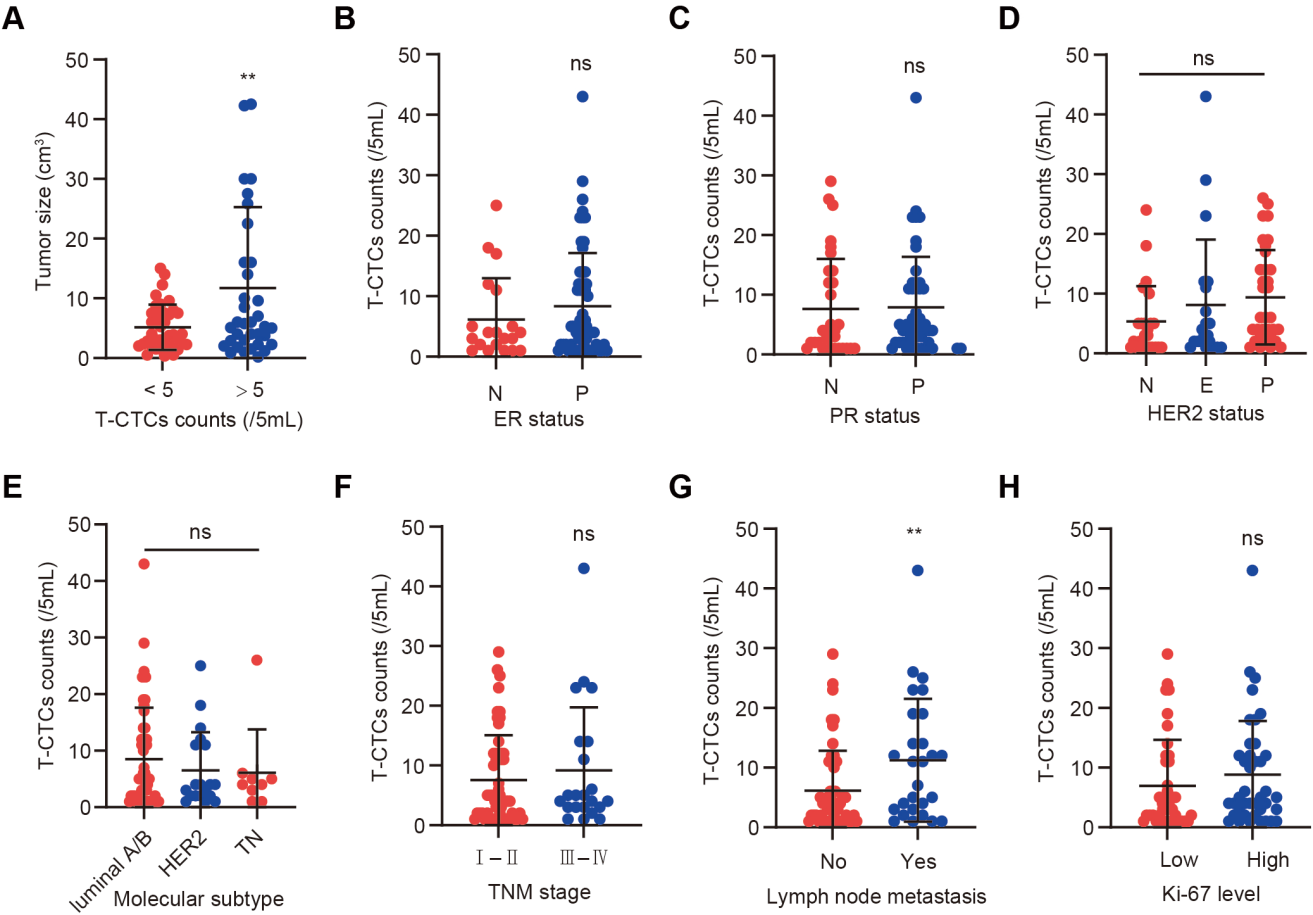
Distribution of T-CTC counts across BC clinical and pathological subgroups. **(A)** Comparison of T-CTC levels across different tumor sizes; **(B–E)** Comparison of T-CTC levels by ER status, PR status, HER2 status, and molecular subtype (Luminal A/B, HER2-positive, triple-negative); **(F)** Comparison of T-CTC levels across TNM stages; **(G)** Comparison of T-CTC counts between patients with and without lymph node metastasis; **(H)** Comparison of T-CTC levels between low Ki-67 expression (< 30%) and high Ki-67 expression (≥ 30%) groups. N indicates negative; P indicates positive; E indicates HER2-E. Ns indicates no statistically significant difference; **p < 0.01.

To investigate the distribution of CTC counts among patients with BC and their association with clinicopathological features, CTC detection was performed on preoperative peripheral blood samples from 93 patients. The results showed that CTCs were detected in 74 patients (79.6%), with a median count of 3 cells per 5 mL of blood, ranging from 1 to 43 cells per 5 mL. Correlation analyses between CTC counts and clinical characteristics were subsequently conducted, as summarized in **Supplementary Table S2**. Patients were stratified based on whether the total number of CTCs (T-CTCs) was ≥5 cells per 5 mL. T-CTC counts differed significantly among groups with different tumor sizes (*p =* 0.011), suggesting that tumor burden may influence CTC levels (**Figure 2A**). However, no significant associations were found between T-CTCs and other clinical variables, including ER status, PR status, HER2 status, or molecular subtype (*p >* 0.05; **Figures 2B-E**). In terms of TNM staging, patients at stage III-IV exhibited a mean T-CTC count of 9.18 ± 10.56, slightly higher than those at stage I-II (7.54 ± 7.51), although the difference was not statistically significant (*p =* 0.447; **Figure 2F**). Notably, the mean T-CTC count in lymph node metastasis-positive patients was 11.22 ± 10.30, significantly higher than that in patients without lymph node metastasis (6.14 ± 6.69; *p =* 0.0098; **Figure 2G**). In contrast, the difference in T-CTC counts between patients with high and low Ki-67 expression was not statistically significant (*p =* 0.323; **Figure 2H**).

Taken together, these findings indicate that CTC counts are closely associated with tumor size and lymph node metastasis, suggesting that CTC enumeration may serve as a reliable biomarker reflecting tumor burden and potential metastatic risk.

### Distribution of EMT phenotype CTCs across different clinical features of BC

To further elucidate the distribution patterns and clinical relevance of CTCs with distinct EMT phenotypes in patients with BC, a systematic analysis was conducted on CTC-positive individuals (n = 74). EMT-based classification revealed that among these patients, E-CTCs were detected in 64.9% (48/74), H-CTCs in 70.3% (52/74), and M-CTCs in 39.2% (29/74).

**Figure 3:**
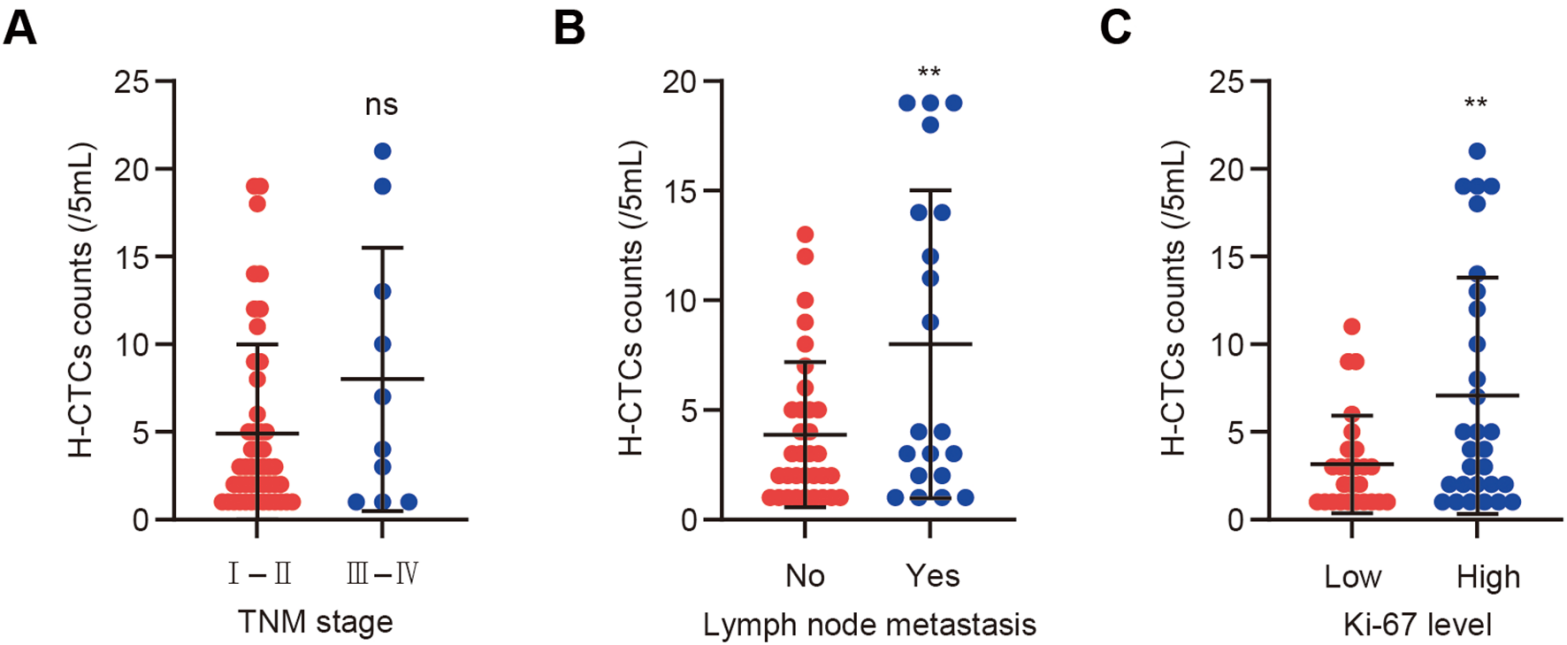
Distribution of H-CTC counts across clinical subgroups of BC patients. **(A)** Comparative analysis of H-CTC levels among patients with different TNM stages; **(B)** Comparison of H-CTC levels between patients with and without lymph node metastasis; **(C)** Comparison of H-CTC levels between low Ki-67 expression (< 30%) and high Ki-67 expression (≥ 30%) groups. Ns indicates no statistically significant difference; **p < 0.01.

Subsequent analyses evaluated the association of each EMT phenotype with TNM stage, lymph node metastasis, and Ki-67 expression level. The number of H-CTCs varied across TNM stages (*p =* 0.335), though the difference was not statistically significant (**Figure 3A**). In contrast, H-CTC counts were significantly higher in patients with lymph node metastasis than in those without (*p =* 0.017), suggesting that H-CTCs may play a key role in metastatic progression (**Figure 3B**). Regarding proliferative activity, patients with high Ki-67 expression exhibited significantly higher H-CTC counts (4.40 ± 6.329) compared to those with low Ki-67 expression (2.02 ± 3.391), with the difference reaching statistical significance (*p =* 0.023; **Figure 3C**). In comparison, no significant differences in E-CTC or M-CTC counts were observed across TNM stage, lymph node status, or Ki-67 expression groups (**Supplementary Table S3**).

In summary, H-CTCs, characterized by both epithelial and mesenchymal features, showed strong associations with lymph node metastasis and Ki-67 expression in BC patients, highlighting their potential clinical value in assessing tumor progression and aggressiveness.

### Distribution of L1CAM-positive CTCs (L1CAM^+^ CTCs) across clinical subgroups of BC

To further investigate the expression profile and clinical relevance of L1CAM in CTCs, L1CAM expression was assessed in 74 BC patients with CTC positivity. The results showed that L1CAM-positive expression in CTCs was detected in 53 patients (71.6%), indicating a relatively high expression rate of L1CAM among CTCs in BC. Specifically, 41 patients (55.4%) exhibited L1CAM expression in H-CTCs; 25 patients (33.8%) showed L1CAM positivity in E-CTCs; and 18 patients (24.3%) had L1CAM expression in M-CTCs.

**Figure 4:**
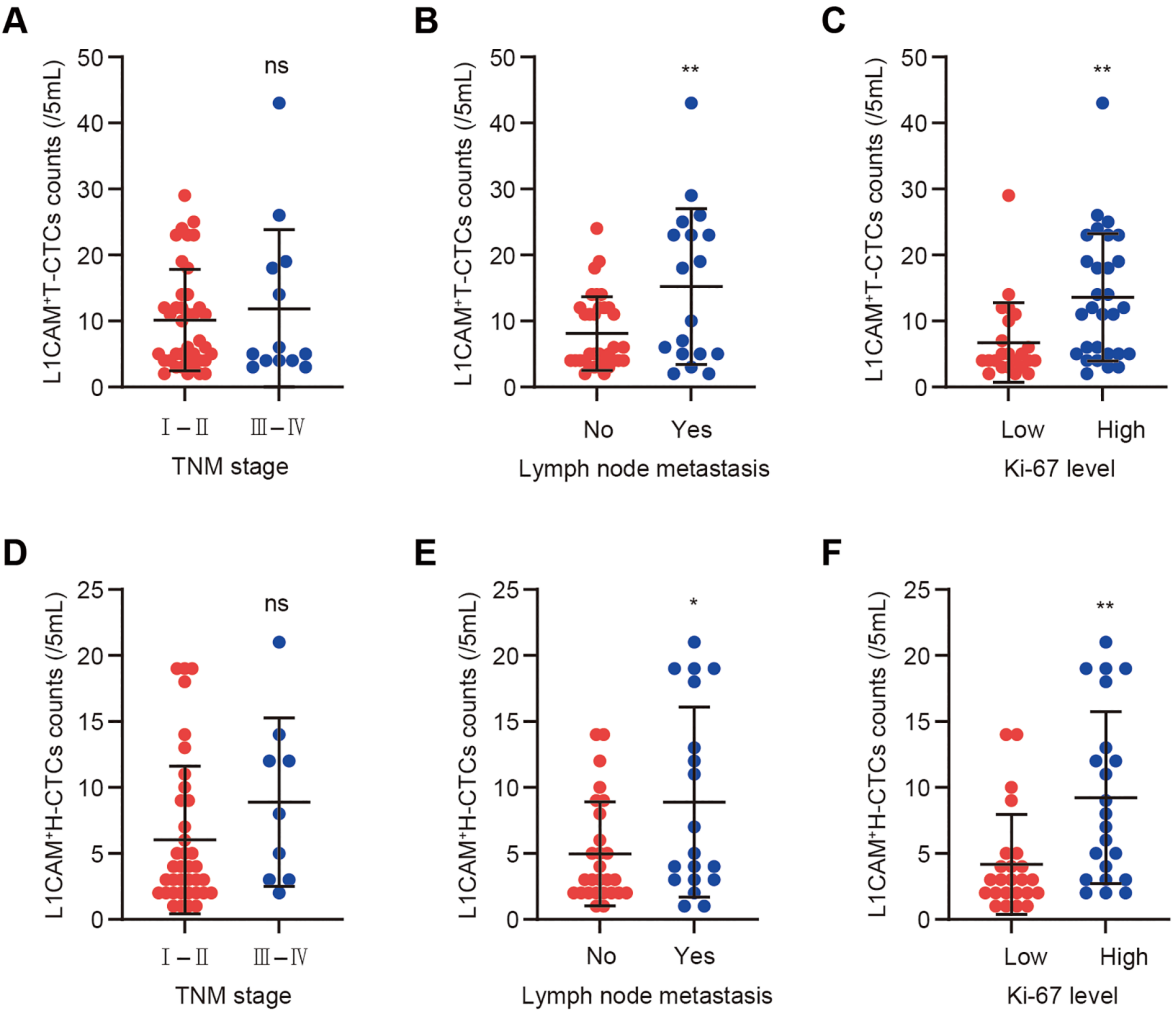
Comparison of L1CAM^+^ CTC counts among BC clinical subgroups. **(A–C)** Comparison of L1CAM^+^ T-CTCs between patients with TNM stage I-II vs. III-IV, with and without lymph node metastasis, and between high and low Ki-67 expression groups; **(D-F)** Comparison of L1CAM^+^ H-CTCs across the same clinical stratifications. For Ki-67, low expression was defined as < 30% positive tumor cell nuclei, and high expression as ≥ 30%. Ns indicates no statistically significant difference; **p < 0.01, *p < 0.05.

The results of the clinical correlation analysis are presented in **Supplementary Table S4**. No significant association was observed between the number of L1CAM-positive T-CTCs (L1CAM^+^ T-CTCs) and TNM stage (**Figure 4A**). However, stratification by lymph node metastasis revealed a significant difference: patients with lymph node metastasis had a mean L1CAM^+^ T-CTC count of 5.05 ± 6.987, which was significantly higher than that of patients without metastasis (2.58 ± 4.849, *p =* 0.048; **Figure 4B**). Additionally, the Ki-67 high-expression group showed a significantly greater number of L1CAM^+^ T-CTCs (5.02 ± 6.989) compared to the low-expression group (2.51 ± 4.726, *p =* 0.042; **Figure 4C**), suggesting that L1CAM^+^ T-CTCs may be associated with tumor cell proliferative activity. Further analysis of L1CAM-positive H-CTCs (L1CAM^+^ H-CTCs) showed no significant correlation with TNM stage (**Figure 4D**). However, a statistically significant difference was observed between patients with and without lymph node metastasis (*p =* 0.043; **Figure 4E**). Moreover, patients with high Ki-67 expression had significantly higher counts of L1CAM^+^ H-CTCs (3.38 ± 5.918) than those with low Ki-67 expression (1.25 ± 3.076, *p =* 0.027; **Figure 4F**).

In summary, the positivity rate of L1CAM in BC CTCs, particularly the high positivity rate in H-CTCs, was closely associated with aggressive tumor features such as lymph node metastasis and elevated Ki-67 index. These findings suggest that L1CAM+ H-CTCs may serve as a potential blood-based biomarker for tumor metastatic potential and biological activity.

### Analysis of factors influencing lymph node metastasis in BC

To further investigate the potential factors influencing lymph node metastasis in BC patients, a multivariate logistic regression analysis was performed based on six variables: CTC phenotypes (E-CTCs, H-CTCs, and M-CTCs), TNM stage, L1CAM expression, and Ki-67 levels. The analysis revealed that H-CTCs (odds ratio [OR] = 1.279, 95% confidence interval [CI]: 0.590-2.539, *p =* 0.0068), L1CAM expression (OR = 8.372, 95% CI: 3.882-17.350, *p =* 0.0124), and Ki-67 levels (OR = 4.636, 95% CI: 1.243-10.140, *p =* 0.0292) were significantly associated with the presence of lymph node metastasis. M-CTCs showed a borderline association (OR = 0.511, 95% CI: 0.138-1.187, *p =* 0.0529), suggesting potential predictive value. In contrast, E-CTCs (*p =* 0.7856) and TNM stage (*p =* 0.0768) did not exhibit statistical significance in this univariate analysis (**Supplementary Table S5**).

Taken together, these findings indicate that elevated H-CTC counts, positive L1CAM expression, and high Ki-67 levels may serve as potential risk factors for lymph node metastasis in BC patients.

### Predictive performance of a combined H-CTCs, L1CAM, and Ki-67 model for lymph node metastasis in BC

**Figure 5:**
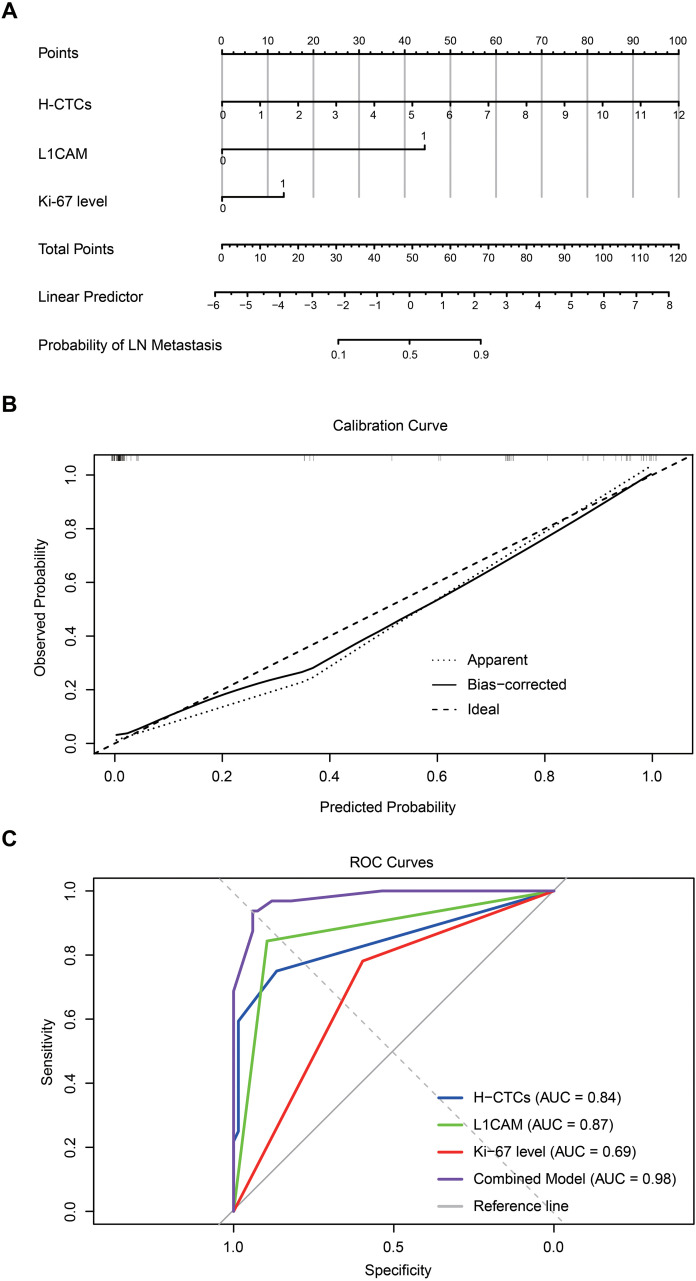
Risk factor prediction for lymph node metastasis in BC patients. **(A)** Nomogram model for quantitative risk assessment of lymph node metastasis in BC; **(B)** Calibration curve of the predictive model; **(C)** ROC curve analysis.

To further quantify the risk of lymph node metastasis in BC patients, a nomogram model was developed based on multivariate logistic regression analysis, incorporating three variables: H-CTC count, L1CAM expression status, and Ki-67 level. The results showed that in the nomogram, the score axes for H-CTCs and L1CAM were substantially longer, indicating that these two variables contributed most significantly to the prediction of metastatic risk. A higher H-CTC count was associated with an increased probability of lymph node metastasis, and positive L1CAM expression also markedly elevated the risk. In contrast, Ki-67 made a relatively smaller contribution to the model’s predictive power (**Figure 5A**). The model yielded a concordance index (C-index) of 0.980, and the calibration curve demonstrated strong agreement between the predicted and observed probabilities, indicating excellent model performance (**Figure 5B**). Receiver operating characteristic (ROC) curve analysis further validated the discriminative ability of the combined predictive model, with an area under the curve (AUC) of 0.98, significantly outperforming each predictor. Specifically, the AUCs for L1CAM and H-CTCs were 0.87 and 0.84, respectively, reflecting high predictive efficacy, whereas Ki-67 showed a relatively limited predictive value with an AUC of 0.69 (**Figure 5C**).

In summary, the nomogram model integrating H-CTC count, L1CAM expression, and Ki-67 level demonstrates excellent accuracy and discriminative capability in predicting lymph node metastasis in BC, highlighting its promising clinical utility, particularly for preoperative individualized risk assessment and therapeutic decision-making.

## Discussion

CTCs have emerged as promising non-invasive “liquid biopsy” biomarkers with significant potential in predicting metastatic risk in breast cancer (23, 24). Previous studies have predominantly focused on the association between CTC counts and prognosis; however, enumeration alone fails to capture the complex biology of tumors and often neglects phenotypic heterogeneity (25, 26). In this study, CTCs were classified into EMT subtypes using the CanPatrol system, and the expression of the molecular marker L1CAM was assessed to systematically evaluate their relationship with lymph node metastasis in breast cancer.

The findings demonstrated that H-CTCs represented the predominant subtype, accounting for 70.3% of CTC-positive samples, and were significantly associated with lymph node metastasis and high Ki-67 expression. L1CAM positivity was detected in 71.6% of peripheral blood CTCs, primarily localized to H-CTCs, and served as an independent predictor of lymph node metastasis (OR ≈ 8.37). A predictive model integrating H-CTC counts, L1CAM positivity, and Ki-67 status achieved excellent performance, with both the C-index and AUC reaching 0.98, markedly outperforming any single variable.

These findings are both consistent with prior observations and significantly innovative. H-CTCs, which simultaneously retain epithelial adhesion and mesenchymal migratory features, are considered the most metastasis-prone subpopulation (27). They have been strongly linked to poor prognosis in lung and colorectal cancers (28, 29), yet systematic analyses in breast cancer remain scarce. While earlier work indicated that ≥ 6 CTCs/5 mL and hybrid/mesenchymal phenotypes correlate with unfavorable progression-free survival (30), our study highlights the dominance of H-CTCs and the prognostic significance of L1CAM positivity, particularly in relation to lymph node metastasis and proliferative index (Ki-67).

Differences in positivity rates across studies mainly stem from detection thresholds and methodological variations: our study adopted ≥ 1 CTC/5 mL as the positivity criterion (positivity rate 79.6%), whereas Xu et al. (30) used ≥ 6 CTCs/5 mL (positivity rate 55.6%). Variations also arose from enrichment strategies (erythrocyte lysis plus nanomembrane vs. Ficoll density gradient) and patient-stage composition (stage I-II dominated in this study vs. stage I-IV in Xu et al.). Nevertheless, both studies converge on the conclusion that higher CTC burden correlates strongly with invasive and metastatic potential.

Importantly, our work revealed for the first time the high expression of L1CAM within CTCs, especially H-CTCs, and its incorporation into a combined predictive model. It significantly enhanced predictive accuracy, extending prior insights that EMT-state CTCs are closely related to tumor aggressiveness, chemoresistance, and adverse outcomes (31). Moreover, recent evidence suggests that targeting EMT processes or selectively eliminating H-CTCs could represent novel therapeutic strategies (32), underscoring the translational implications of our findings.

H-CTCs, characterized by concurrent epithelial adhesion and mesenchymal migratory capabilities, exhibit enhanced adaptability and survival during hematogenous dissemination, which underlies their high metastatic potential (27). Previous evidence has demonstrated strong correlations between H-CTCs and poor outcomes in lung and colorectal cancers (28, 29), and our study confirms similar trends in breast cancer, showing significant associations with both lymph node metastasis and Ki-67 overexpression, consistent with earlier observations (33).

This study also provides the first systematic assessment of L1CAM expression in peripheral blood CTCs in breast cancer, with a positivity rate of 71.6%, predominantly localized to H-CTCs. L1CAM, previously implicated in EMT, migration, and stemness properties across various malignancies (34, 35), demonstrated strong predictive value here (OR ≈ 8.37). Mechanistically, it may promote integrin-mediated adhesion signaling and activate the FAK/ERK pathway, thereby enhancing CTC motility, invasiveness, and resistance to hostile microenvironments (16, 36).

Ki-67 is widely established as a proliferative marker in breast cancer pathology (37, 38). We found that patients with high Ki-67 expression exhibited significantly elevated H-CTC and L1CAM+ CTC counts, suggesting that proliferative activity may facilitate CTC release and survival in circulation. By integrating H-CTCs, L1CAM, and Ki-67, our model achieved an AUC of 0.98, surpassing the performance of existing nodal prediction models that largely rely on imaging or histopathological indicators (AUC 0.80-0.90) (39, 40). Given its non-invasive nature and capacity for dynamic monitoring, this model shows strong potential for preoperative risk stratification, occult nodal metastasis detection, neoadjuvant therapy response evaluation, and recurrence surveillance (41). Comparable literature also suggests that hybrid EMT-state CTCs readily form microemboli, increasing circulatory survival and facilitating distant metastasis (42, 43), reinforcing the central role of H-CTCs and L1CAM in metastatic dissemination.

This study is limited by its single-center, retrospective design, relatively small cohort, and predominance of early-stage patients, which may constrain generalizability. The enrichment strategy (erythrocyte lysis plus nanomembrane filtration) could introduce methodological bias in EMT subtype distribution. Additionally, the predictive model has not undergone internal optimism correction or external validation, raising the possibility of overfitting. Furthermore, clinical variables such as tumor size, grade, and lymphovascular invasion were not fully incorporated, and survival outcomes (DFS/PFS/OS) were not analyzed. Reliable CTC detection remains challenging given their rarity and lack of absolute specific markers (44, 45).

Future studies should involve large-scale, multicenter prospective cohorts with longitudinal follow-up to validate the stability and prognostic value of H-CTCs and L1CAM. Incorporating decision curve analysis, bootstrap validation, and DeLong testing will further refine the robustness and clinical benefit of predictive models. Mechanistic studies are also warranted to elucidate L1CAM’s functional role in CTC biology and evaluate its therapeutic potential, alongside integration of immune microenvironmental features to construct more comprehensive models for breast cancer metastasis monitoring and intervention.

## Conclusion

This study systematically analyzes the distribution of EMT subtypes of CTCs and their L1CAM positivity profiles in patients with ESIBC, revealing a close relationship between phenotypic heterogeneity of CTCs and tumor biological behavior. Specifically, L1CAM+ H-CTCs are significantly associated with lymph node metastasis, while L1CAM+ M-CTCs correlate with PR status, suggesting their potential involvement in tumor micrometastasis and hormone receptor-related pathways. ROC analysis further indicates that the combination of H-CTCs, L1CAM+ M-CTCs, and Ki-67 markedly improves the accuracy of predicting lymph node metastasis. Therefore, EMT-based CTC subtypes characterized by L1CAM positivity not only hold promise as biomarkers but also provide novel perspectives for molecular subtyping and personalized management of BC.

## Data Availability

The original contributions presented in the study are included in the article/**Supplementary Material**. Further inquiries can be directed to the corresponding author.

## Supplementary material

Supplementary Figure 1 Overview of study design and analytical strategy.

Supplementary Figure 2 Fluorescence microscopy images of different CTC subtypes. Blue fluorescence: DAPI; red fluorescence: epithelial marker; green fluorescence: mesenchymal marker signal; purple fluorescence: L1CAM gene expression.
