# Supplementary material for: Evaluating metastatic risk in breast cancer through CTCs and L1CAM expression
Source: Front Oncol. 2025 Nov 11;15:1686166. doi: 10.3389/fonc.2025.1686166 (PMC12643865; doi:10.3389/fonc.2025.1686166)
Supplement: Supplementary file 1 [file Table1.docx]

**Table S1. Clinical and pathological features of enrolled patients.**

| **Feature** | **Breast cancer patients (n=93)** |
| --- | --- |
| Age (years) | 51 (28-82) |
| Tumor size (cm^3^) | 8.35 ± 10.32 |
| TNM staging (%) | Stage I-II: 81 (87.10) Stage Ⅲ-Ⅳ: 12 (12.90) |
| ER (%) | P: 68 (73.12) N: 25 (26.88) |
| PR (%) | P: 54 (58.06) N: 39 (41.94) |
| HER2 (%) | P: 42 (45.16) N: 24 (25.81) |
|  | E: 27 (28.72) |
| Molecular subtype (%) | luminal A/B: 58 (62.36) |
|  | HER2: 26 (27.96) |
|  | TN: 9 (9.68) |
| Ki-67 | 31.67 ± 20.86 |
| Lymph node metastasis | Yes: 32 (34.41) |
|  | No: 61 (65.59) |

Note: P, Positive; N, Negative; E, Equivocal; TN, triple-negative breast cancer.

**Table S2. Distribution of T-CTCs in different clinical characteristic groups.**

| **Feature** | **T-CTCs (n=74)** | ***p*-value** |
| --- | --- | --- |
| Tumor size (cm^3^) | ≥5/5 mL: 11.72 ± 13.56 | 0.0054 |
|  | <5/5 mL: 5.15 ± 3.81 |  |
| ER (%) | P: 6.82 ± 8.83 N: 6.15 ± 8.33 | 0.3162 |
| PR (%) | P: 7.89 ± 8.46  N: 7.62 ± 8.39 | 0.8885 |
| HER2 (%) | P: 9.38 ± 7.91 N: 5.36 ± 5.92 | 0.1881 |
|  | E: 8.09 ± 10.97 |  |
| Molecular subtype (%) | luminal A/B: 8.53 ± 9.04 | 0.5527 |
|  | HER2: 6.52 ± 6.76 |  |
|  | TN: 6.11 ± 7.65 |  |
| TNM staging (%) | Stage I-II: 7.54 ± 7.51 | 0.447 |
|  | Stage Ⅲ-Ⅳ: 9.18 ± 10.56 |  |
| Lymph node metastasis | Yes: 11.22 ± 10.30 | 0.0098 |
|  | No: 6.14 ± 6.69 |  |
| Ki-67 | High: 8.80 ± 9.01 | 0.3239 |
|  | Low: 6.92 ± 7.72 |  |

Note: P, Positive; N, Negative; E, Equivocal; TN, triple-negative breast cancer.

**Table S3. Distribution of EMT-associated CTC subtypes in relation to clinical characteristics.**

| **Feature** | **H-CTCs (n=52)** | ***p*-value** | **E-CTCs (n=52)** | ***p*-value** | **M-CTCs (n=52)** | ***p*-value** |
| --- | --- | --- | --- | --- | --- | --- |
| TNM staging (%) |  |  |  |  |  |  |
| Stage I-II: | 4.90 ± 5.09 | 0.1134 | 9.00 ± 8.11 | 0.9366 | 2.00 ± 1.10 | 0.2202 |
| Stage Ⅲ-Ⅳ: | 8.00 ± 7.51 |  | 9.25 ± 13.13 |  | 3.67 ± 3.19 |  |
| Lymph node metastasis |  |  |  |  |  |  |
| Yes: | 8.00 ± 7.02 | 0.0056 | 4.35 ± 6.20 | 0.2405 | 3.25 ± 2.44 | 0.9036 |
| No: | 3.88 ± 3.31 |  | 2.40 ± 1.99 |  | 3.40 ± 3.18 |  |
| Ki-67 |  |  |  |  |  |  |
| High: | 7.07 ± 6.74 | 0.0080 | 4.32 ± 7.36 | 0.4991 | 3.24 ± 2.84 | 0.9124 |
| Low: | 3.15 ± 2.78 |  | 3.30 ± 2.52 |  | 3.36 ± 3.27 |  |

Note: P, Positive; N, Negative.

**Table S4. Distribution of L1CAM⁺CTCs and L1CAM⁺H-CTCs across clinical subgroups.**

| **Feature** | **L1CAM⁺T-CTCs (n=53)** | ***p*-value** | **L1CAM⁺H-CTCs (n=41)** | ***p*-value** |
| --- | --- | --- | --- | --- |
| TNM staging (%) | | | | |
| Stage I-II: | 10.13 ± 7.67 | 0.5498 | 6.03 ± 5.59 | 0.1867 |
| Stage Ⅲ-Ⅳ: | 11.85 ± 12.02 |  | 8.89 ± 6.37 |  |
| Lymph node metastasis | | | | |
| Yes: | 15.22 ± 11.81 | 0.0046 | 8.90 ± 7.20 | 0.0216 |
| No: | 8.09 ± 5.58 |  | 4.96 ± 3.94 |  |
| Ki-67 | | | | |
| High: | 13.59 ± 9.63 | 0.0045 | 9.23 ± 6.50 | 0.0021 |
| Low: | 6.74 ± 6.02 |  | 4.17 ± 3.77 |  |

Note: P, Positive; N, Negative.

**Table S5. Univariate logistic regression analysis of factors associated with lymph node metastasis.**

| **Variables** | **Univariate analysis** | |
| --- | --- | --- |
|  | **OR (95% CI)** | ***p*-value** |
| E-CTCs | 0.063 (0.323-0.639) | 0.7856 |
| H-CTCs | 1.279 (0.590-2.539) | 0.0068 |
| M-CTCs | 0.511 (0.138-1.187) | 0.0529 |
| TNM staging | 0.130 (0.009-1.076) | 0.0768 |
| L1CAM | 8.372 (3.882-17.350) | 0.0124 |
| Ki-67 level | 4.636 (1.243-10.140) | 0.0292 |
