# Supplementary figures and images for: Evaluating metastatic risk in breast cancer through CTCs and L1CAM expression
Source: Front Oncol. 2025 Nov 11;15:1686166. doi: 10.3389/fonc.2025.1686166 (PMC12643865; doi:10.3389/fonc.2025.1686166)

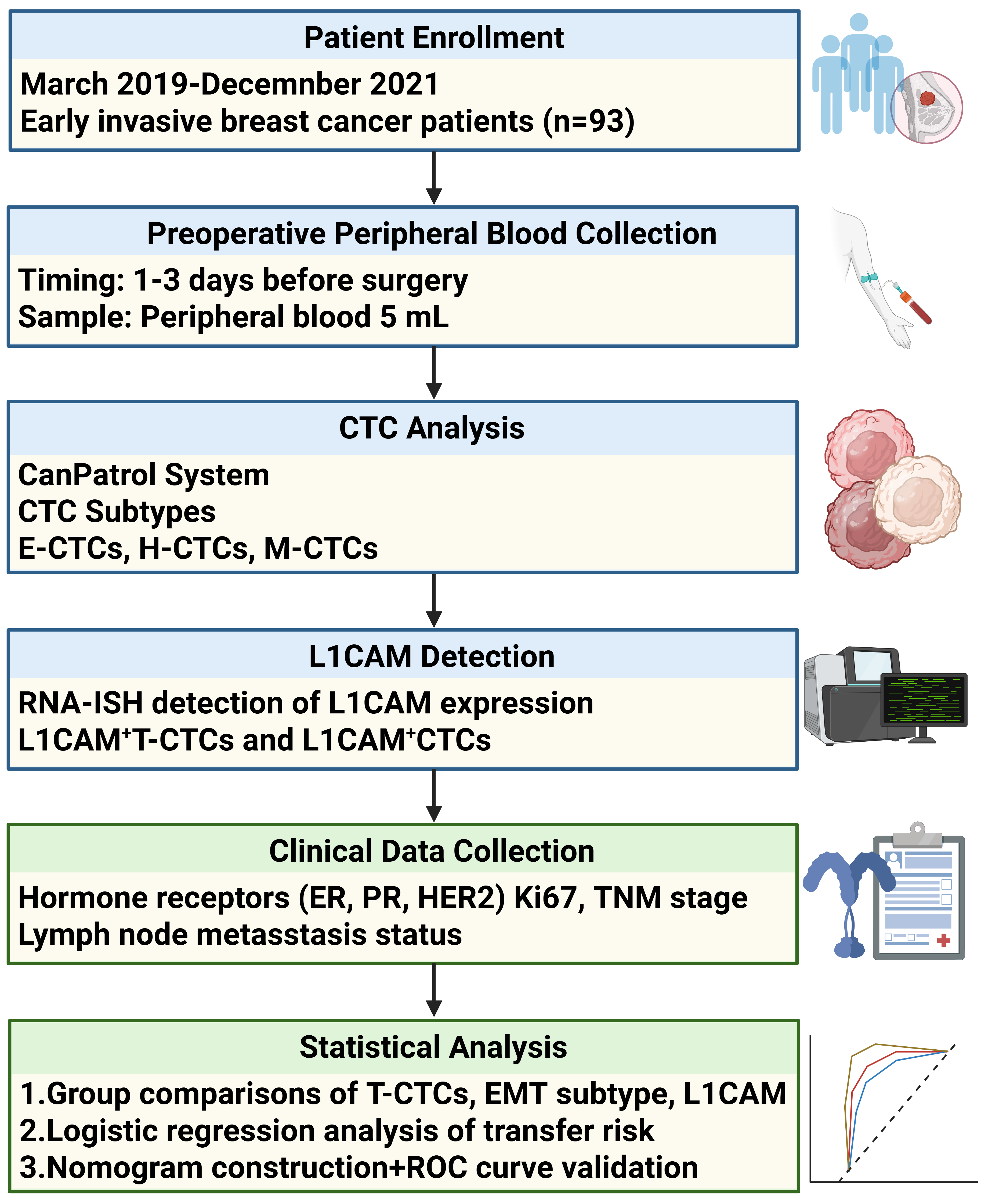

Supplement: Supplementary Figure 1 — Overview of study design and analytical strategy. [file Image1.jpeg]

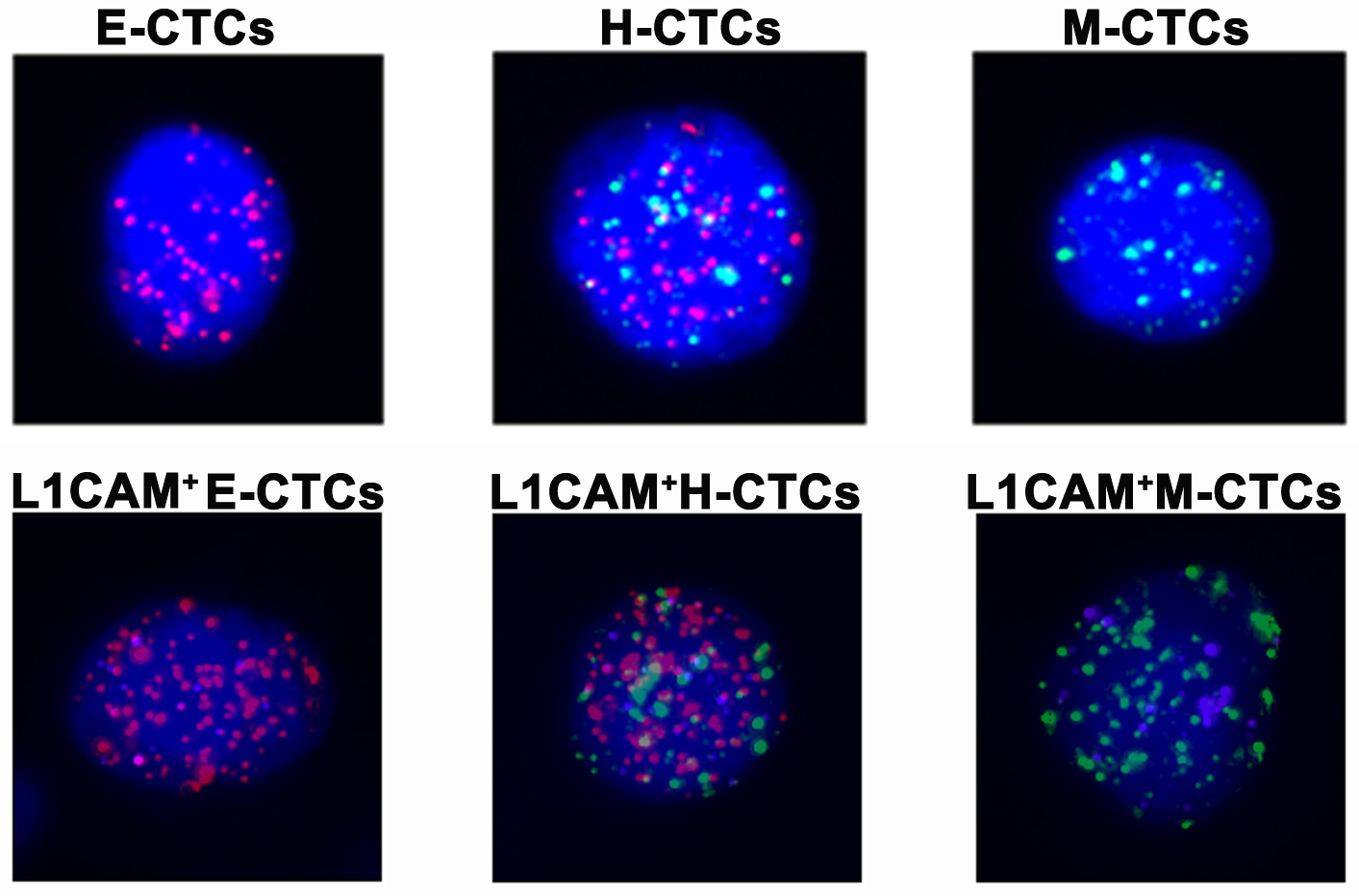

Supplement: Supplementary Figure 2 — Fluorescence microscopy images of different CTC subtypes. Blue fluorescence: DAPI; red fluorescence: epithelial marker; green fluorescence: mesenchymal marker signal; purple fluorescence: L1CAM gene expression. [file Image2.jpeg]
